# Supplementary material for: Evaluation of deep convolutional neural networks for in situ hybridization gene expression image representation
Source: PLoS One. 2022 Jan 24;17(1):e0262717. doi: 10.1371/journal.pone.0262717 (PMC8786163; doi:10.1371/journal.pone.0262717)
Supplement: Attachment — Submitted filename: Review response 1 (1).pdf [file pone.0262717.s003.pdf]

We thank both reviewers for their thoughtful comments and efforts towards improving our manuscript. We have submitted the revised version and a separate copy with changes tracked for your convenience.

> Reviewer #1: The authors have proposed an approach for embedded
> representation learning from ISH images. Overall, the embedded feature
> space has been demonstrated for its relevance to molecular features and
> disease prediction such as schizophrenia. The authors have further
> illustrated it in the context of medical informatics and molecular
> genetics. The experiments look enough for PLOS CB. I have few
> suggestions:
> 1. As the submitting journal is PLOS CB, it could be nice for the authors
> to compare their approach with other possible methods. In particular, the
> embedded space should be evaluated in a competitive manner.

This manuscript is no longer being considered to PLOS CB. Correspondingly, we believe this suggestion is of lower importance to the readers.

For this work, our primary goal wasn’t to compare methods but instead to introduce this dataset and the gene re-identification approach. We feel that comparing several methods will distract from this goal. We hope that readers will extend our work by testing new methods for generating embeddings. However, we do competitively compare our embedding space to a base ResNet. Furthermore, we have experimented with using SIFT descriptors as embeddings in the Allen Mouse Brain Atlas and found they were inferior. This agrees with the proven effectiveness of deep convolutional neural networks in other domains.

> 2. The embedded space is claimed to provide good features for disease
> prediction. I was wondering the difficulty of the proposed disease
> prediction problem. Is there any way to illustrate that the prediction is
> challenging or easy to be classified?

Schizophrenia disease prediction from brain images is challenging. We used experimental factors and demographic variables to demonstrate this. Moreover, it is known that schizophrenia is very heterogeneous and difficult to diagnose. We have added text to our limitations paragraph to mention this challenge:
“In the context of predicting diagnoses, we note that schizophrenia is a very heterogeneous disorder (Huber 1997). The difficulty of identifying schizophrenia from brain images evident from a recent MRI study that reported an AUC of 0.71 in a held-out test dataset (Oh et al. 2020).“

However, we were primarily interested in detecting postmortem molecular signals of schizophrenia to aid the understanding of disease processes.

> 3. The resolution of the patches looks pretty low. What is the utility of
> it? Why did the authors choose such low-resolution ?

We agree that this resolution is low and discuss this in the limitations paragraph of our Discussion:
“Regarding scale, our input patches of 65 thousand pixels capture less than 1% of the original image resolution. As deep learning tools improve, we aim to test our approach on finer images.”
This is primarily a technical limitation and we look forward to better hardware and methods that can handle higher resolution. This limit, which is mirrored across image processing broadly, is somewhat reduced by our sampling of 50 patches per image.

> 4. The authors have focused on ROC. How about the PRC curves and its
> area?

We agree and have added area under the precision-recall curve statistics to Table 2 and Supplement Table 1. We note that the values in these tables have changed slightly due to the needed revaluation of the classifiers. We also briefly comment on those values in the Results section. We did not similarly add this metric to the GO membership prediction section because of the donor-specific signals we detected.

> 5. Such study is vulnearble to the underlying image processing steps and
> choices. I wish the authors can elaborate as details as possible for
> those upstream steps.

The use of deep convolutional neural networks has minimized the hand-engineered image processing steps in our work and image processing broadly. This is why work greatly contrasts the previous analyses of these images by the Allen Institute for Brain science. Specifically, those previous analyses used complex processing steps that were designed by hand while we provide a machine learning approach that operates on patches from the base images.

The only processing we do is downsampling and foreground/background segmentation. We additionally perform some augmentation by flipping the images. We also state parameters not explicitly related to image processing, such as batch sizes and learning rates. These are detailed in our methods section. Our publicly provided source code provides a complete and reproducible implementation of these steps.

> 6. Statistical significance has to be ensured throughout all the
> comparisons.

For the rank-1 metrics, we provide comparisons to a random baseline. We believe this is sufficient given the large gap between random and our learned classifiers (13.2 fold increase). We have made this more clear by adding these fold change values to Table 1.

For the prediction of expression level and pattern, we have added Mann Whitney U p-values to Tables 2 and S1. These values show that we are able to predict gene annotations far beyond chance levels.

We did not similarly add this metric to the GO membership prediction section because the donor-specific signals we detected negated the usefulness of this task.

In our last Results section, we have included paired t-test statistics for the relevant comparisons.

> Reviewer #2: The author applied DCNN and triplet loss function to learn
> the representation of ISH image in human brain for the gene
> re-identification. They also applied learned embedding to diagnose
> schizophrenia in another dataset. There are several major concerns about
> the manuscript:
> 1. Do the manually selected 40 random regions at Cortex study affect
> the performance and result?

We do not describe 40 random regions. The misunderstanding might be due to our selection of 40 random images for manual annotation of the foreground. These annotated foregrounds are used to detect the foreground in the remaining images. The foreground segmentations are then used to extract random patches or regions from the full-size images. We consider the foreground segmentation step to be minor and its effects on performance to be negligible. We have edited the text in this part of the Methods section to make this more clear.

> Not sure why the author will choose the
> different dimensions in random patches compare to the training.

We converted the full-size images to 224x224 pixels for learning the foreground segmentation masks. This was for convenience as it was the default setting in the segmentation tool we used.

From the full images, we extracted random patches that were sized 256x256 pixels. This mirrored the settings for the triplet-reid tool we used for this stage of the study.

So, while they are different dimensions they are used for different steps that operate at different levels (full image versus patch). The resolution was constant across the training, validation and test split to ensure correct comparisons.

We have added the following text in the Methods section to clarify this:
"This resolution differs from the foreground mask stage due to the different tools used, but we note they are operating at different levels (image versus patch). For both stages, the resolution is constant across the training, test and validation sets. "

> 2.   It is really suspicious that for the gene re-identification, the
> performance of random, ResNet, and the "Triplet Loss" at training <
> validation < test set. Usually, the performance should be reversed. If it
> is due to the randomness of the data split, then at least three-fold
> cross validation and average across split should be utilized.. Moreover,
> the model applied to new dataset showed better performance, this is also
> unusual.

We agree that upon first glance, these results would seem suspicious. However, the increase in performance is due to the sets containing a smaller set of genes that are to be reidentified. So the training dataset contains ~800 genes, and the test and validation sets include roughly 100. In this case, even a random classifier will have a higher accuracy when operating in a set of 100 genes in comparison to 800. This is reflected in the first row of Table 1, which displays the random rank-1 accuracy scores.

To prevent readers from overlooking this detail, we have edited both the text and Table 1. In Table 1, we have added the fold increase over random in parentheses. In the Results text, we have added the following:
"The rank-1 accuracy progressively increases across the training, validation and test sets. This performance trend is the reverse of the expectation of an accuracy drop in unseen datasets. However, because we are testing gene re-identification, the number of unique genes in the splits partially determines the accuracy. To account for these differences in size, we compare the metrics to the random baseline, decreases from training to test (from 102 times the random performance to 13.2, Table 1)."

> 3.   For the gene re-identification, it seems that the labels are
> unbalanced, so the author should use F1 and precision-recall curve rather
> than AUC for the major evaluation.

For gene re-identification, we use rank-1 accuracy, which is a primary metric for person re-identification and image classification broadly. Imbalance is not a concern for this strict metric that only examines nearest neighbours in the embedding space. Also, the F1 metric would not be applicable as it would require setting a threshold on distance in the embedding space, which was not learned in the gene re-identification stage. While

other metrics are used for re-identification tasks, they are not as applicable to the dataset as rank-1 accuracy is because of donor-specific biases in the dataset.

Guided by your and Reviewer 1's request we have added area under the precision-recall curve and F1 statistics to the GO analysis results. This information is now included in Tables 2 and S1. We note that the values in these tables have changed slightly due to the needed revaluation of the classifiers. As newly mentioned in the text, examination of these metrics did not change our interpretation.

> 4. In the section of using learned embedding to predict expression
> level. "This suggests information about the region assessed for
> expression is not a key indicator for gene re-identification". This
> really challenges the current popular view in the spatial transcriptome.

Neuroanatomically, the cerebral cortex is referred to as the 'isocortex' because of its consistent laminar structure that appears in both the temporal and visual cortex. Spatial transcriptomics usually assays expression in a single cortical region across this laminar architecture. These studies then describe expression patterns in areas such as layer 1 or layer 5. We show that this level of spatial detail is in our embeddings in support of this current popular view. In contrast, the quoted sentence refers to differences between the temporal and visual cortices. Like Zeng et al., we found few differences between these two cortical regions. For example, a layer 2 marker gene would mark layer 2 across the cortical regions. I believe that a spatial transcriptomics study would also find expression patterns for any given gene to be similar in the temporal and visual cortices. We have edited the quoted sentence to clarify that we are referring to two cortical brain regions:

"Although limited to the temporal or visual cortices, this suggests information about the region assessed for expression is not a key indicator for gene re-identification."

In summary, gene expression varies at the cytoarchitectural scale but less so at the broader visual versus temporal cortex scale. This aligns with current views of the spatial transcriptome.

> 5. In the section to diagnose schizophrenia, the ResNet showed even
> worse performance than the random. Moreover, the Demographic variable
> showed better performance than embedding and the combination of embedding
> and demographic. These indicated that the conclusion in this section are
> totally just due to the batch effect (tissue ph, age..), which should not
> be listed as the main achievement in this paper.

We agree and do not list this as the main achievement.

We do not consider the demographic variables to represent a batch effect. For example, it is known that smoking is more common in individuals with schizophrenia. The limited amount of postmortem data makes it difficult to obtain matched data. This is not a batch effect. Additionally, batch information is not available for this dataset. We tested classifiers with both gene expression and these additional factors to assess their effect. We found that there was a marginal improvement for some genes and state this as:

“These genes provide only a marginal performance improvement, suggesting the expression patterns that discriminate individuals with schizophrenia mostly reflect differences due to age, pH or smoking status.“

If requested, we are happy to rephrase a specific piece of text relating to this concern.
